# A large pellet size rather than diet complexity increases piglet creep feed disappearance and feeding activity but not growth performance

**DOI:** 10.1093/tas/txaf114

**Published:** 2025-08-27

**Authors:** Malene Hald, Tina Skau Nielsen, Thomas Sønderby Bruun, Kristian Knage-Drangsfeldt, Trine Friis Pedersen

**Affiliations:** Department of Animal and Veterinary Sciences, Aarhus University, Campus Viborg, DK-8830 Tjele, Denmark; Department of Animal and Veterinary Sciences, Aarhus University, Campus Viborg, DK-8830 Tjele, Denmark; SEGES Innovation, DK-8200 Aarhus N, Denmark; Vestjyllands Andel A.m.b.a, DK-7400 Herning, Denmark; Department of Animal and Veterinary Sciences, Aarhus University, Campus Viborg, DK-8830 Tjele, Denmark

**Keywords:** diet complexity, feed disappearance, growth performance, pellet size, piglet creep feed

## Abstract

The objective of the study was to investigate how creep feed pellet size and diet complexity influenced piglets’ pre-weaning feed consumption and eating behavior, as well as pre- and post-weaning (PW) growth performance. In total, 180 litters were allocated to one of six dietary treatments: positive control (commercial crumble feed, PCON; *n*=30 litters), negative control (no creep feed provided; NCON: *n*=30 litters), high complexity large pellets (HCLP; *n*=30 litters), low complexity small pellets (LCSP; *n*=30 litters), low complexity large pellets (LCLP; *n*=30 litters), or bakery meal large pellets (BMLP; *n*=30 litters) offered from day 8 in the lactation period until weaning. Large pellets (LP) had a diameter of 12 mm and a length of 10-40 mm, whereas small pellets (SP) had a diameter of 2 mm and a length of 5-15 mm. The term “high or low complexity” of diets refers to the choice of ingredients included; both diets were wheat-based, but the high-complexity diet included more digestible protein sources and functional additives such as aromas and probiotics. Piglets from 36 litters (PCON; *n*=6 litters, NCON; *n*=6 litters, HCLP; *n*=6 litters, LCSP; *n*=6 litters, LCLP; *n*=6 litters, and BMLP; *n*=6 litters) were recorded individually during the lactation period until 15 days PW. The litters and piglets were weighed on days 0, 8, and 15 of lactation and at weaning on day 21. In addition, the individually monitored piglets were also weighed at days 9 and 15 PW. Creep feed disappearance was measured in litters with individually monitored piglets during the entire creep feeding period, as were behavioral observations (on days 9, 16, and 19 of lactation), to assess how many piglets in each litter had their head in the feeder or feed items in their mouth. Dietary treatment had no effect on pre-weaning BW, BW gain, or ADG (*P*>0.05). However, piglets fed large pellets (HCLP, LCLP, and BMLP) showed a significantly (*P*<.0001) higher feed disappearance than piglets fed PCON and LCSP, which was supported by a higher number of piglets observed eating on days 16 and 20 of lactation for these treatments (*P*=0.001 and <0.001, respectively). Post-weaning, piglets fed PCON had a 40% numerically higher ADG compared with piglets fed NCON. In conclusion, the physical form of the creep feed affected the piglets’ feeding activity, although this was not reflected in their growth performance. Also, diet complexity did not seem to impact growth performance when the piglets were weaned on day 21 of lactation.

## Teaser

Larger pellets increased both feed disappearance and feeding activity in piglets before weaning, suggesting that physical form may play a key role in encouraging early feed intake.

## INTRODUCTION

In modern pig production, increasing the litter size of sows is an important strategy to optimize both productivity and profit. Danish sows currently give birth to an average of 18.2 liveborn piglets per litter (Hyttel., 2024), although most sows are unable to rear more than 14 piglets until weaning due to limitations in the number of functional teats. An increased litter size reduces the average piglet birth weight (Roehe, 1999; Riddersholm et al., 2021; Johnson et al., 2022). Furthermore, a large litter size also increases the risk of poor piglet growth rates if litters are not standardized to meet the number of functional teats in the sow because more piglets may miss milk letdowns (Ocepek et al., 2017; Kobek-Kjeldager et al., 2020). Although a sow’s milk yield increases as she nurses more piglets (Auldist et al., 1998), the distribution of milk among the piglets results in less milk available per piglet, leading to a decline in individual piglet growth (Bruun et al., 2017) (. In addition, lower milk availability can also lead to higher mortality, possibly due to low birth weight piglets spending more time near the sow’s udder, thereby increasing the risk of crushing (Weary et al., 1996).

Some, but far from all, previous studies indicate that creep feeding during lactation can increase the weaning weight and that it is positively correlated with the amount of solid feed consumed during the initial post-weaning (PW) period (Aherne et al., 1982; Bruininx et al., 2002; Kuller et al., 2007; Sulabo et al., 2010; Lee and Kim, 2018). The amount of solid feed intake is particularly important, since one of the major challenges in the transition from sow’s milk to cereal-based creep feed is the significant decrease in nutrient and energy intake. This results in a critical period of underfeeding, in which the piglet’s energy requirements may not be met until several days PW (Le Dividich and Sève, 2000). However, a piglet’s individual creep feed consumption is difficult to measure, although it is generally considered to be low, with intake varying significantly both within and between litters (Pajor et al., 1991; Bruininx et al., 2002, 2004; Pluske et al., 2007).

The daily creep feed intake is influenced by several factors, including litter size and age of the piglets, but also by the physical form of the feed and the dietary ingredients (complexity) included (Okai et al., 1976; Barnett et al., 1989; Pajor et al., 2002; Klindt, 2003; van den Brand et al., 2014; Clark et al., 2016; Middelkoop et al., 2019). The physical form of the creep feed can be characterized by pellet size, with most creep feed measuring 2-3 mm in diameter (Edge et al., 2005). However, some studies indicate that piglets may find larger pellets more attractive (van den Brand et al., 2014; Clark et al., 2016; Middelkoop et al., 2018; Craig et al., 2021) and that larger pellets may stimulate more feed-directed behavior compared with smaller pellets (Edge et al., 2005; van der Meulen et al., 2010; Middelkoop et al., 2018). Creep feed offered to piglets is typically highly complex in composition, meaning that highly digestible animal protein sources such as milk powder, whey powder, fish meal, blood plasma, or refined vegetable protein sources such as soy protein concentrate and potato protein concentrate are included to a larger extent than soybean meal. The major protein source used in low complexity diets is typically soybean meal and to a lesser extent protein sources of animal origin and refined vegetable protein sources (Fraser et al., 1994; Mahan et al., 2004; Skinner et al., 2014). Flavor, for example sweetness of the creep feed, may also be a factor that could influence feed intake (Middelkoop et al., 2019).

The objective of the current study was to investigate the effect of physical form and diet complexity on piglets’ creep feed disappearance, feed behavior, and growth during the lactation period and on PW performance. It was hypothesized that piglets offered creep feed in the form of large pellets would show more feeding behavior directed towards solid feed, eat more, and perform better than piglets offered small pellets, crumble, or no creep feed. The complexity of the diet was hypothesized to have less of an impact on these parameters than the physical characteristics of the feed.

## MATERIAL AND METHODS

### Ethics Approval

The animal experimental procedures and care of animals were performed in accordance with the Danish laws and regulations for the humane care and use of animals in research (Act no. 1107 of 1/7/2022, Ministry of Food, Agriculture, and Fisheries of Denmark, 2020) and under consideration of the Arrive Guidelines (Du Sert et al., 2020).

### Animals and Housing

The experiment was carried out from July to September 2023 in a commercial pig herd in the western part of Denmark. The herd consisted of 1,900 DanBred Landrace × DanBred Yorkshire sows, which were all inseminated with DanBred Duroc semen (Hatting Agro, Horsens, Denmark). The sows were moved to the farrowing unit approximately five days before expected farrowing. The farrowing units were equipped with farrowing pens (2.6 × 1.7 m) with metal crates (ACO Funki A/S, Herning, Denmark) to protect the piglets from being crushed. The temperature was set at 21 °C. Each farrowing pen was equipped with a covered nest area (1.18 × 0.9 × 0.3 m) containing a heating panel (Aniheater, Furturefarming Aps., Holsted, Denmark) and floor heating for the piglets. The nest area had a temperature of 30-35 °C at farrowing. The nest area was provided with heat-treated straw daily. In two of the farrowing units, the floor in the farrowing pens was a fully slatted plastic floor with cast iron slats under the sow, and five other farrowing units had a partially slatted floor (1/3 cast iron slatted floor and 2/3 concrete floor). The sow was fed three times daily at 0600, 1200, and 2359 hours. Sows and piglets had ad libitum access to water throughout the experimental period. The PW pens were 4.58 × 2.35 m (2.58 × 2.35 m solid floor, 2.00 × 2.35 m slatted floor) with a covered nest area measuring 2.35 × 1.35 m including the concrete floor heating. At weaning, piglets were mixed when moved to the weaning facility and were sorted by size independently of the dietary treatment in the farrowing unit.

Over a period of nine weeks, a total of 180 litters from 2^nd^ to 6^th^ parity sows were included in the experiment from parturition until weaning at day 21. Both sows nursing their own litter (non-nurse) and sows nursing a new litter after having nursed their own piglets for seven to eight days (nurse sow) were included. A subgroup of 489 piglets from 36 litters (six litters from each dietary treatment) were ear tagged (Merko Smågris with number, ALLFLEX dan-mark ApS, Lemvig, Denmark) with individual numbers so that the individual piglet´s performance could be monitored during the lactation period, and at days 9 and 15 PW. Sows included in the experiment were randomly selected before farrowing based on sow parity. Non-nurse and nurse sows were standardized to a litter size of 14 piglets, regardless of the number of functional teats, within 12 to 24 hours after birth to ensure sufficient colostrum intake before excess piglets were moved. Large piglets were moved to nurse sows, and the remaining piglets were standardized with non-nurse sows according to size by removing the smallest piglets within each litter. Nurse sows were selected from among sows that had farrowed seven to eight days earlier. Piglets allocated to a nurse sow were offered milk replacer for the first two days after being moved.

### Dietary Treatments and Feeding

**Figure 1.**
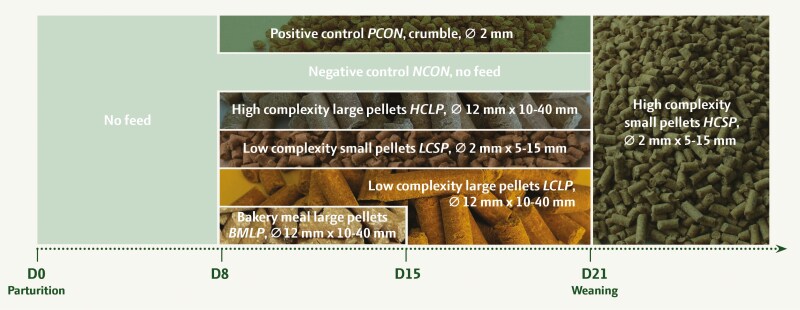
Overview of the six treatments, including their complexity, physical form (⌀ refers to the diameter and the interval refers to the length of the pellets), and the duration of the creep feed period.

Piglets were assigned to one of six dietary treatments, which varied in physical form (Fig. 1). The physical forms were: large pellets (diameter 12 mm, length 10-40 mm), small pellets (diameter 2 mm, length 5-15 mm), and crumble (diameter 2 mm). The ingredient composition was either high or low complexity or sweet taste. In this study, the term “complexity” refers to the inclusion of high-digestibility ingredients. The high complexity treatment included highly digestible protein sources such as whey powder, fish meal, and soy protein concentrate compared with the low complexity diet, which contained protein sources of both lower digestibility (soybean meal) and digestible sources to a lesser extent, for example soy protein concentrate. Lastly, bakery meal, a combined product of 90% cake and dough residues from the bakery industry containing 10% whey powder concentrate, was applied in one of the dietary treatments to provide a sweet taste. The high complexity treatment also contained aroma in the form of a commercial flavor (not specified) and vanilla flavor and cheese powder. The treatments were: 1) Positive control (PCON), where the piglets were given a commercial crumble creep feed selected as the preferred feed by the herd manager, 2) negative control, where the piglets did not receive creep feed during the lactation period (NCON), 3) a high complexity diet processed into large 12-mm pellets (HCLP), 4) a low complexity diet pressed into 2-mm pellets (LCSP), 5) a low complexity diet processed into large 12-mm pellets (LCLP), and 6) a sweet-tasting diet processed into large 12-mm pellets (bakery meal large 12-mm pellets; BMLP). After weaning, the piglets were fed with the high complexity diet consisting of 2-mm pellets (HCSP) with the same dietary composition as HCLP. Treatments will henceforth be referred to by their abbreviations. Piglets receiving the BMLP treatment were offered the sweet-tasting BMLP from days 8 to 14 during the lactation period to stimulate them to eat more before changing to LCLP from day 15 until weaning to increase the nutritional value in terms of dietary CP. The list of ingredients and the chemical composition of the treatments are presented in Table 1, except for the PCON treatment, since detailed information on this diet could not be obtained from the company.

**Table 1.** Dietary ingredients, chemical composition, and calculated nutrient content of the dietary treatments

Item	Positive control^1^	Low complexity^2^		High complexity^3^		Bakery meal^4^
	Crumble	2-mm pellets	12-mm pellets	2-mm pellets	12-mm pellets	12-mm pellets
**Ingredients, %**						
Wheat	X	45.5		30.5		
Wheat flakes	X					
Wheat, popped				30.0		
Barley	X	20.0				
Corn flakes	X					
Soybeans, toasted	X					
Soybean meal, dehulled toasted	X	7.00				
Soy protein concentrate	X	5.73		8.50		
Oats, dehulled		5.00		7.00		
Bakery meal		2.00		3.00		90.0
Lactose	X	2.00		3.00		
Sugar	X					
Potato protein concentrate				2.00		
Cheese powder		2.00		2.00		
Skimmed milk powder	X					
Egg powder		2.00		1.00		
Sugar beet molasses		1.78		1.95		
Fish meal		1.00		2.00		
Whey powder	X			1.50		
Whey powder concentrate						10.0
Yoghurt milk				1.50		
Palm oil	X					
Rapeseed oil	X	0.40		0.75		
Sunflower oil	X					
Fish oil				0.10		
Premix	X	5.605^5^		5.206^6^		
**Planned chemical composition (as is)**						
Dry matter, %	-	87.0		89.2		89.2
Crude protein, g/kg	178	165		175		15.8
Crude fat, g/kg	40	43.5		45.3		106
Crude ash, g/kg	50	51.9		48.9		13.8
SID^7^ lysine, g/kg	10.9	12.0		12.7		10.6

^1^Positive control = PCON, quantitative information on the main ingredients and nutrient content could not be obtained from the company.

^2^High complexity = HCLP.

^3^Low complexity = LCSP and LCLP.

^4^Bakery meal = BMLP.

^5^Containing vitamins, minerals, amino acids, antioxidants, acids, and enzymes.

^6^Containing vitamins, minerals, amino acids, according to Danish Nutrient Standards (Tybirk et al., 2024) and furthermore provided unspecified acids, probiotics, enzymes, aroma, toxin binder, and antioxidants.

^7^Standardized ileal digestibility.

The PCON was produced by DENKAVIT (Duo Krumme, Voorthuizen, the Netherlands) for Nutrax ApS (Støvring, Denmark). The HCLP, LCLP, and LCSP were produced by Vestjyllands Andel A.m.b.a (Herning, Denmark) at their feed mill (Hee, Ringkøbing, Denmark), whereas the production of the BMLP diet and re-pelleting of the large pellets for HCLP, LCLP, and BMLP were carried out at Adival (Billund, Denmark).

Piglets were fed ad libitum in round (diameter 28 cm) or elongated troughs (26.7×10 cm) from day 8 post parturition until weaning (except for piglets in the non-feeding treatment group, NCON). From days 8 to 14, the piglets were fed twice daily, with the first feeding taking place between 0600 and 0800 hours and the second feeding between 1300 and 1400 hours. From day 14 until weaning, the piglets were fed three times daily, with the first feeding taking place between 0600 and 0800 hours, the second feeding between 1000 and 1100 hours, and the third feeding between 1300 and 1500 hours. The amount of creep feed offered to the piglets was adjusted to correspond to the disappeared amount.

### Measurements

#### Feed disappearance.

The feed disappearance was estimated for a subset of litters receiving creep feed. For each of the 29 litters (attributed to the five treatment groups receiving creep feed), a bucket was placed in the corner of each farrowing pen, and when the piglets had been fed, the same amount of feed was placed in the bucket. The bucket was weighed at the same time every day from day 9 (one day after the start of feeding) until weaning.

#### Piglet performance.

All piglets (both litter weight and individual weight) were weighed at days 0, 8, 15, and at weaning. Piglets with ear tags were weighed individually at the same four times mentioned above and also on days 9 and 15 PW. All weighings were performed using a digital scale (Bjerringbro Vægte ApS, Bjerringbro, Denmark).

#### Eating behavior.

**Figure 2.**
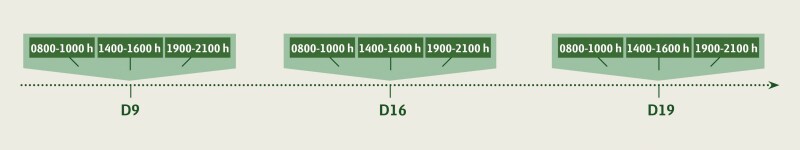
Timeline for recording eating behavior on days 9, 16, and 19 of lactation. Each litter was observed every 4.5 minutes over a 2-hour period, three times per observation day.

Live behavioral observations were conducted by a single observer in a subsample of litters for the piglets receiving creep feed. The number of piglets eating was recorded for each litter on days 9, 16, and 19. “A piglet eating” was defined as a piglet with its head in the feeding trough or a piglet clearly observed with creep feed in its mouth. The method used was an instantaneous scan sampling, with scans every 4.5 minutes over three 2-hour periods on days 9, 16, and 19 during the lactation period (Fig. 2).

#### Management.

Sows were offered straw as nest building material, and they were also offered wrap silage in the pen on a daily basis as rooting material. Piglets were not castrated. Four to six days after parturition, piglets were tail docked (approx. 1/3) to prevent tail biting in the growing period. Medical treatments of sows and piglets followed normal practice in the herd.

### Calculations

Average daily gain and BW gain were calculated for litters and individual piglets from the day of birth during lactation until weaning, and for 15 days PW for individual piglets.

The number of piglets per litter eating on days 9, 16, and 19 was calculated as the total number of piglets observed eating from all 16 scans on days 9, 16, and 19. The total number of piglets eating in a litter was calculated as the number of piglets out of the total number of observed piglets eating at every scan on days 9, 16, and 19 (16 scans × three days). An estimate for the maximum gain retained from the ingestion of creep feed was calculated as the total SID lysine concentration (g/kg) in the feed multiplied by the amount of consumed feed (kg) divided by the retained amount of Lys/kg gain (adapted from NRC (2012)):


$$\begin{array}{l} Estimated\;litter\;gain\;from\;feed,\%=\frac{\;\left(\frac{SID\;Lys\;concentration\;in\;the\;feed\left(\frac{g}{kg}\right)\times feed\;consumed\;\left(kg\right)}{19\;g\;SID\;Lys\;per\;kg\;gain}\right)}{Total\;litter\;gain\;\left(kg\right)}*100 \end{array}$$

### Statistical Analyses

Data from the experiment were analyzed as four different datasets: one dataset for litter data (*n* = 180 litters), one dataset for individual piglets (*n* = 489 piglets), one dataset for feed disappearance (*n* = 29 litters), and one dataset for eating behavior (*n* = 29 litters). The data were analyzed using the MIXED procedure in SAS (version 9.4, SAS Institute Inc., Cary, NC).

The BW change of the average piglet in the litter, the litter BW change, litter gain, and the ADG of the average piglet in the litter in the periods from days 1 to 8, 9 to 15, and 16 until weaning, and eating behavior were tested using the following models.

Data on the percentage of piglets with their head in the feed trough or clearly observed with feed in their mouth were analyzed using the following model:


$$\begin{array}{l} Y_{ij}=\mu +\alpha_{i}+\beta_{j}+\gamma_{k}+{\left(\alpha \beta \right)}_{ij}+\varepsilon_{ijk} \end{array}$$

where $Y_{ij}$ is the observed trait, μ is the overall mean of observations, $\alpha_{i}$ is the main effect of dietary treatment (i = 1, 2, 3, 4, and 5), $\beta_{j}$ is the main effect of day (j = 9, 15, and 20), $\gamma_{k}$ is the main effect of section (j = slatted floor or partially slatted floor), ${\left(\alpha \beta \right)}_{ij}\;$is the effect of the interaction between day and treatment, and $\varepsilon_{ijk}$ is the residual random component.

The litter feed disappearance was analyzed using the following model:


$$\begin{array}{l} Y_{ij}=\mu +\alpha_{i}+\beta_{j}+{\left(\alpha \beta \right)}_{ij}+\varepsilon_{ij} \end{array}$$

where $Y_{ij}$ is the observed trait, μ is the overall mean of observations, $\alpha_{i}$ is the main effect of dietary treatment (i = 1, 2, 3, 4, and 5), $\beta_{j}$ is the main effect of day (j = day 8, 9, 10, …. 23), ${\left(\alpha \beta \right)}_{ij}$ is the interaction between treatment and day and $\varepsilon_{ij}$ is the residual random component.

Litter performance was analyzed using the following model:


$$\begin{array}{l} Y_{ijklmno}=\mu +\alpha_{i}+\beta_{j}+\gamma_{k}+\delta_{l}+\nu_{m}+\rho_{n}+\varrho_{o}+{\left(\alpha \beta \right)}_{ij}+{\left(\alpha \nu \right)}_{im}+{\left(\beta \nu \right)}_{jm}+\varepsilon_{ijklmno} \end{array}$$

where $Y_{ijklmno}$ is the observed trait, μ is the overall mean of observations, $\alpha_{i}$ is the main effect of dietary treatment (i = 1, 2, 3, 4, 5, and 6), $\beta_{j}$ is the effect of day during the lactation period (j = 0, 8, 15, 21, and 22), $\gamma_{k}$ is the main effect of parity (k = 2, 3, 4, 5, and 6), $\delta_{l}$ is the main effect of housing units (l = slatted floor or partially slatted floor), $\nu_{m}$ is the effect of sow type (m = non-nurse sow or nurse sow), $\rho_{n}$ is the effect of weaning age (day) as a covariate (n = 16, 17, 18, 19, 20, 21, 22, 23, and 24), $\varrho_{o}$ is the random effect of sow (o = 1, 2, 3 … 167), ${\left(\alpha \beta \right)}_{ij}$ is the interaction between treatment and day, ${\left(\alpha \nu \right)}_{im}$ is the interaction between treatment and nurse sow, ${\left(\beta \nu \right)}_{jm}$ is the interaction between day and nurse sow, and $\varepsilon_{ijklmno}$ is the residual random component. No three-way interaction between sow type, day, and treatment was observed for any of the variables (*P* > 0.05), and it was therefore omitted in the final model. To account for repeated measurements within each litter and individual piglet, day during the lactation period was included as a repeated measurement.

The individual piglet data, including BW, weight gain, and ADG, were analyzed using the following model:


$$\begin{array}{l} Y_{ijklmn}=\mu +\alpha_{i}+\beta_{j}+\gamma_{k}+\delta_{l}+\nu_{m}+\;\rho_{n}+{\left(\alpha \beta \right)}_{ij}+\varepsilon_{ijklmn} \end{array}$$

where $Y_{ijklm}$ is the observed trait, μ is the overall mean of observations, $\alpha_{i}$ is the main effect of dietary treatment (*i* = 1, 2, 3, 4, 5, and 6), $\beta_{j}$ is the effect of day during lactation (*j* = 0, 8, 21, and 22), $\gamma_{k}$ is the main effect of parity (*k* = 2, 3, 4, 5, and 6), $\delta_{l}$ is the main effect of housing section (l = slatted floor or partially slatted floor), $\nu_{m}$ is the effect of nurse sow (*m* = non-nurse sow or nurse sow), $\rho_{n}$ is the effect of weaning age (day) as a covariate (*n* = 20, 21, 22, and 23), ${\left(\alpha \beta \right)}_{ij}$ is the interaction between treatment and day, and $\varepsilon_{ijklmn}$ is the residual random component. To account for repeated measurements for each piglet, day during the lactation period was included as a repeated component.

To account for multiple mean comparisons, *P*-values were adjusted using a Tukey-test. Statistical significance was accepted when *P* < 0.05, and tendencies were accepted at *P* ≤ 0.10. Mean values are presented as least-square means (LSMEANS) together with the highest value of the SEM. Outliers were identified by evaluating residual plots and removed when the residuals were more than three times higher than the respective SEM. The floor type and feed trough were accounted for in the statistical handling of data as a combined fixed effect called section. Litter BW, the average piglet BW and feed disappearance were log-transformed before being analyzed in the model. The means were back-transformed by taking the exponential function of the log-transformed value.

## RESULTS

Thirteen sows/litters (2, 2, 2, 4, 1, 2 from treatments PCON, NCON, HCLP, LCSP, LCLP and BMLP, respectively) were excluded from the dataset due to decreased nursing capacity caused by mastitis, metritis, or agalactia. Furthermore, one sow (LCSP) was weaned early and was thus excluded due to a shoulder ulcer.

### Eating Behavior, Feed Disappearance and Litter Gain from Feed

No treatment effect was observed on the percentage of piglets with their head in the feeding trough or with feed in their mouth on day 9 (Table 2). On observation days 9, 15, and 19, the interaction of treatment and day affected the piglets’ eating behavior (*P* < 0.05). On observation days 15 and 19, the LCLP and BMLP treatments resulted in a higher percentage of piglets observed with their head in the feeding trough or eating creep feed compared with the PCON, HCLP, and LCSP treatments (*P* < 0.05).

**Table 2.** Percentage of piglets in a litter observed with their head in the feeding trough or with feed in their mouth, on observation days 9, 15, and 19 during the lactation period.

	Treatment^1^						P-values			
	PCON	HCLP	LCSP	LCLP	BMLP	SEM	Trt^2^	Day	Section^3^	Trt^2^*day
n litters	6	6	5	6	6	-				
**Eating behavior, %**							< 0.0001	< 0.0001	0.89	0.0005
Day 9	2.98	6.40	6.56	14.2	8.35	4.29				
Day 15	6.80^b^	12.5^b^	6.85^b^	37.5^a^	39.4^a^	4.42				
Day 19	8.90^b^	11.5^ab^	6.44^b^	36.5^a^	26.2^a^	4.43				

Values are presented as least squares treatment means with the SEM.

^a,b^Means with different superscript letters within day differ significantly (*P* < 0.05).

^1^Control (PCON), high complexity large 12-mm pellets (HCLP), low complexity pellets (LCSP), low complexity large 12-mm pellets (LCLP), and bakery meal large 12-mm pellets (BMLP).

^2^Treatment.

^3^Section: sow and litters housed on either slatted or partially slatted floor. The piglets’ feed troughs were different from each other due to the floor type.

**Figure 3.**
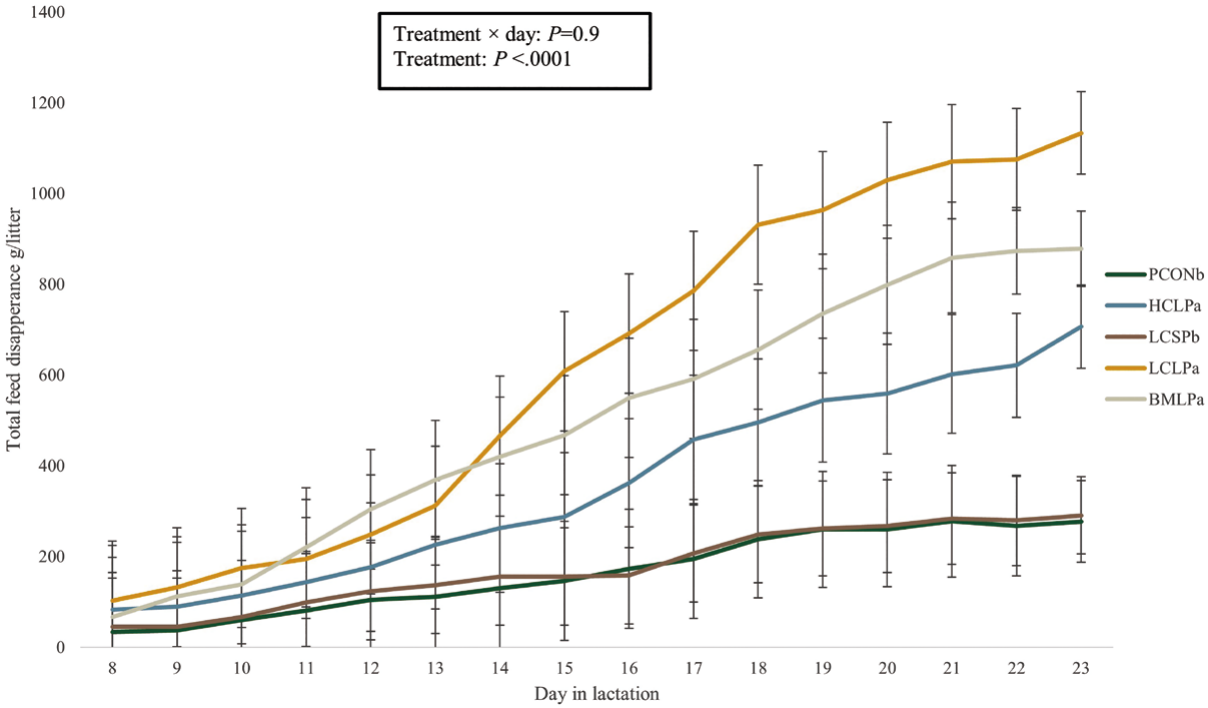
Total feed disappearance for the treatments providing creep feed during the lactation period from day 8 until weaning (N = 35 litters). Control (PCON), high complexity 12-mm pellets (HCLP), low complexity small 2-mm pellets (LCSP), low complexity large 12-mm pellets (LCLP), and bakery meal large 12-mm pellets (BMLP). Values are presented as least-square means, and different superscript letters (a and b) differ significantly (P < 0.05).

**Figure 4:**
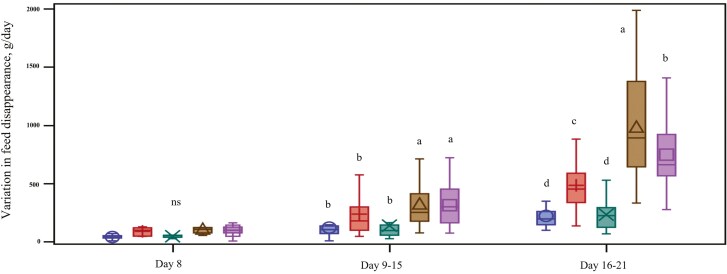
Variations in feed disappearance between litters receiving the same dietary treatment and changes over the observed period (N = 35 litters). The boxplot shows the 25^th^ and 75^th^ percentiles, the dots indicate the mean, and the straight lines represent the median for each treatment. Control (PCON, blue box), high complexity 12-mm pellets (HCLP, red box), low complexity small 2-mm pellets (LCSP, green box), low complexity large 12-mm pellets (LCLP, brown box), and bakery meal large 12-mm pellets (BMLP, purple box). Means with different superscript letters (^a,b,c,d^) within day differ significantly (P < 0.05).

The amount of feed offered to piglets based on the disappearance of feed from troughs was higher for the piglets fed HCLP, LCLP, and BMLP than for piglets fed PCON and LCSP (*P* < 0.05; Fig. 3). Large variations in feed disappearance were found between litters receiving the same dietary treatment, and feed disappearance also changed over the observed period (Fig. 4). The percentage of gain explained by solid feed intake was generally low (less than 3 %) and did not differ between treatments (*P* > 0.05; Table 3). However, litters fed LCLP and BMLP had the numerically highest percentage of gain explained by solid feed intake. From day 14 of lactation until weaning, piglets fed LCLP showed the highest feed disappearance, numerically. The daily amount of creep feed provided was higher for the BMLP piglets when they were fed bakery meal in large pellets and decreased after day 15 when they were fed with LCLP. There were no differences in feed disappearance for the LCSP and HCLP treatments during the lactation period.

**Table 3.** Performance of litters and the average piglet in a litter on days 0, 8, and 15 of lactation and at weaning. The litters were offered one of six dietary treatments from day 8 during the lactation period until weaning.

	Treatment^1^							Sow type			P-value						
	PCON	NCON	HCLP	LCSP	LCLP	BMLP	SEM	Non-nurse	Nurse	SEM	Trt^2^	Day	Parity	Sow type	WA^3^	Day sow type^3^	Trt^2^ Day
No. Litter	28	28	28	26	29	28	-	100	68	-	-	-	-	-	-	-	-
Mean sow parity	3.5	3.4	3.6	3.3	3.7	3.1	-	-	-	-	-	-	-	-	-	-	-
**Litter weight, kg**																	
Day 0	20.0	20.0	18.9	18.7	20.0	19.8	0.76	16.8	22.7	0.59	0.39	<.0001	0.07	<.0001	0.06	0.001	0.93
Day 8	32.2	33.5	30.9	31.7	34.2	33.6	1.30	29.0	36.8	0.96							
Day 15	49.3	52.5	49.6	48.1	53.1	51.3	2.03	45.3	56.8	1.48							
Weaning	65.2	68.7	66.3	63.5	69.0	67.7	2.66	61.3	72.6	1.86							
**Litter weight gain, kg/day**																	
Days 0 to 8	2.00	2.24	1.91	1.98	2.09	2.18	0.21	1.96	2.15	0.19	0.09	<.0001	0.11	0.46	0.12	0.0002	0.29
Days 8 to 15	3.18	3.55	3.02	2.78	3.58	3.17	0.21	2.93	3.44	0.19							
Day 15 to weaning	3.70	3.21	3.03	2.91	3.29	3.18	0.30	3.29	2.96	0.19							
Day 0 to weaning	2.96	3.00	2.66	2.56	2.98	2.84	0.14	2.79	2.88	0.18							
**Gain from feed, %**																	
Entire lactation	0.37	-	0.70	0.30	1.07	2.59	0.69	-	-	-	0.10	-	0.97	0.89	0.95	-	-
**ADG per average piglet in the litter, g/day**																	
Days 0 to 8	144	162	139	144	151	157	0.02	143	156	0.01	0.15	<.0001	0.02	0.43	0.16	0.001	0.16
Days 8 to 15	268	285	256	231	288	253	0.02	247	280	0.01							
Day 15 to weaning	319	270	266	261	273	274	0.02	280	290	0.01							
Day 0 to weaning	244	239	221	212	237	227	0.01	227	233	0.01							

Values are presented as least-square means with the SEM.

^1^ Control (PCON), negative control (NCON), high complexity large 12-mm pellets (HCLP), low complexity small 2-mm pellets (LCSP), low complexity large 12-mm pellets, and bakery meal large 12-mm pellets (BMLP)

^2^Treatment

^3^Weaning age

### Individual Piglet Performance

There was an interaction between treatment and day on individual piglet BW, BW gain, and ADG (*P < *0.01; Table 4). Piglets fed large pellets showed a numerically higher ADG than piglets receiving the LCSP, PCON, and NCON treatments from d 15 to weaning. From day 15 until weaning, there was a numerically higher ADG (+ 19%) for piglets fed LCLP compared with LCSP, even though the nutrient composition of the two treatments were identical. Although not significant, piglets from all treatments experienced a 70-72% decrease in ADG from the time of weaning to day 9 PW compared with the period from day 15 of lactation until weaning. From day 9 PW and day 15 PW, piglets fed with PCON and HCLP had the numerically highest ADG, and the NCON piglets had the numerically lowest ADG (38.9% difference between highest and lowest ADG 15 days PW). The weaning age significantly affected (*P* = 0.02) the weaning weight and tended to influence the ADG (*P* = 0.08).

**Table 4.** Individual piglet performance in the lactation period and on days 9 and 15 post-weaning. Dietary treatments were provided from day 8 in the lactation period until weaning.

Item		Treatment^1^ (Trt)					P-value						
	PCON	NCON	HCLP	LCSP	LCLP	BMLP	SEM	Trt^2^	Day	Parity	Section^3^	WA^4^	Trt^2^×Day
**Weight, kg**													
Day 0	1.30	1.16	1.16	1.24	1.21	1.31	0.04	0.24	0.007	< 0.0001	< 0.0001	0.02	< 0.0001
Day 8	2.42	2.51	2.45	2.54	2.41	2.54	0.07						
Day 15	3.74	3.80	3.91	4.01	3.94	3.98	0.11						
Weaning	5.25	5.44	5.48	5.63	5.68	5.70	0.18						
9 days PW	5.66	5.88	5.81	5.97	6.14	6.35	0.21						
15 days PW	7.02	6.83	7.12	7.02	7.28	7.52	0.20						
**Gain, kg**													
Days 0 to 8	1.14	1.43	1.33	1.32	1.25	1.29	0.08	0.44	0.006	< 0.0001	0.009	0.23	0.001
Days 8 to 15	1.35	1.33	1.49	1.51	1.61	1.50	0.08						
Day 15 to weaning	1.54	1.71	1.60	1.65	1.81	1.75	0.08						
Weaning to 9 days PW	0.49	0.44	0.33	0.29	0.36	0.62	0.10						
Weaning to 15 days PW	1.43	0.97	1.36	1.09	1.15	1.23	0.11						
**ADG, g/day**													
Days 0 to 8	142	178	163	166	159	159	0.11	0.61	0.32	< 0.0001	0.02	0.08	<0.001
Days 8 to 15	197	194	210	237	228	216	0.11						
Day 15 to weaning	236	258	251	231	276	273	0.11						
Weaning to 9 days PW	49.1	47.9	34.8	35.6	39.9	56.7	0.14						
Weaning to 15 days PW	215	153	210	167	182	176	0.16						

Values are presented as least-square means with the SEM.

^1^Control (PCON), negative control (NCON), high complexity 12-mm pellets (HCLP), low complexity small 2-mm pellets (LCSP), low complexity large 12-mm pellets (LCLP), and bakery meal large 12-mm pellets (BMLP).

^2^Treatment.

^3^Section: describes that the sow and piglets were housed on either a slatted floor or a partially slatted floor. The piglets’ feed troughs were different from each other due to the floor type.

^4^Weaning age.

### Litter Performance

Total litter weight at days 0, 8, 15, and at weaning, litter weight gain, and average piglet ADG did not differ between dietary treatments or between housing units. Furthermore, no significant interactions between treatment and day and between treatment and nurse sow were found (*P* > 0.05; Table 3). Litters fed LCLP had the numerically highest litter weight at weaning (69.0 kg), but it was only 300 g greater than for NCON litters, which did not receive creep feed. From days 8 to 15 of the lactation period, the average piglet in litters fed LCLP had a numerically higher ADG compared with average piglets from all other treatments except for the NCON piglets, whose ADG (285 g/d) was similar to that of the LCLP average piglets (288 g/d). Piglets offered LCSP had a numerically lower ADG during the entire lactation period compared with piglets offered LCLP, although the ingredients and nutrient composition of the two treatments were identical.

Litter weight was higher at days 0, 8, 15, and at weaning for litters raised by a nurse sow compared with a non-nurse sow (*P* < 0.001). However, litter weight gain and piglet ADG during the entire lactation period did not differ between litters reared by nurse sows compared with piglets reared by non-nurse sows (*P* = 0.46; *P* = 0.43, respectively). Piglets reared by nurse sows had a significantly higher ADG than piglets reared by non-nurse sows (*P* = 0.001).

## DISCUSSION

### Pellet Size Effect on Feed Disappearance and Growth Performance

It was expected that offering creep feed to piglets would increase litter gain and weaning weight. Furthermore, it was expected that piglets offered large pellets would have a higher feed disappearance and show more eating behavior compared with piglets fed small pellets or crumble. However, there was no effect of dietary treatments on growth performance throughout the entire lactation period, although feed disappearance was greater and piglets showed more eating behavior in the groups fed with large pellets (HCLP, LCLP, and BMLP) compared with the PCON, LCSP, and NCON groups. The fact that offering creep feed did not improve piglet performance in the lactation period is in line with other studies (van den Brand et al., 2014; Clark et al., 2016; Middelkoop et al., 2018; Craig et al., 2021), whereas some studies successfully increased piglet gain when providing creep feed (Fraser et al., 1994; Lee and Kim, 2018). The great variability in performance outcome attributed to creep feeding is most likely caused by multiple factors. The interaction between treatment and day was most likely caused by the increased eating behavior and numerically higher feed disappearance when piglets were fed large pellets compared with small pellets or crumble. However, it is not known if it is the same or different piglets that contribute to the percentage, since the individual piglets were not marked or given an identification number in the present experiment. With regard to pellet size, the present study indicates that large pellets were more efficient at stimulating feed intake and eating behavior as well as feed disappearance compared with small pellets or crumble. However, the increased feed disappearance did not manifest itself as increased weaning weight or higher piglet ADG during lactation. Furthermore, previous studies have demonstrated that larger pellets increase creep feed intake compared with small pellets (Edge et al., 2005; van den Brand et al., 2014; Clark et al., 2016; Middelkoop et al., 2018; Craig et al., 2021). In two experiments, van den Brand et al. (2014) observed no difference in feed intake when piglets were fed large or small pellets after days 11 and 17 of lactation. This could indicate that the preference for large pellets is most pronounced in early lactation, although the present study did not confirm this based on the results for feed disappearance. However, the interaction between treatment and day on BW, BW gain, and ADG indicates that pellet size and diet complexity (treatment) affect the piglets differently at different time points, which was also observed for eating behavior (% piglets eating). In the present study, the mean total feed disappearance was 657 g during the feeding period of 13 days (approximately 50 g per litter per day). Bruininx et al. (2002) and Middelkoop et al. (2020) reported that 65 to 74% of total creep feed intake occurs from days 21 to 30. The most commonly observed feed intake ranges from 5 to 60 g per piglet per day throughout the entire lactation period, with intakes generally increasing as lactation progresses (Fraser et al., 1994; Bruininx et al., 2002; Kuller et al., 2010; Sulabo et al., 2010; Yan et al., 2011b, 2011c; Adeleye et al., 2014; Heo et al., 2018; Isensee et al., 2020). Adeleye et al. (2014) estimated that a daily intake of approximately 60 g creep feed per piglet during the lactation period can increase the piglet’s gain by 1 kg during the first two weeks PW. Given the variability in creep feed intake and the common feed intake range, the lack of any effect on subsequent growth may be caused by weaning at day 21 in the present study, which prevented a high proportion of piglets from adapting to solid feed intake as lactation progressed. It might be speculated that the higher feed disappearance using large pellets would have been even more pronounced if the piglets had been weaned at day 28 due to the larger number of piglets eating and also a generally higher pre-weaning feed intake, which ultimately could have affected ADG, weaning weight, and subsequent ADG.

It would have been interesting to investigate which categories of piglets (e.g., low birth weight, limited teat access, or slow growth) benefit most from creep feeding. However, this was not possible, since individual nutritional status, feed intake, and visits to the creep feeder were not assessed. Variability in milk intake due to teat order (for example, posterior teats produce lower quality milk than anterior and middle teats (Pluske et al., 2007; Skok et al., 2007; Pedersen et al., 2011)) may have a greater impact than the individual consumption of creep feed. The variability in milk intake within a litter and between litters might explain the difference in solid feed intake within litters. Pajor et al. (1991) expected to see a high creep feed intake by small piglets compared with large piglets due to increased motivation and stated the ‘motivation or compensatory feeding hypothesis’. Several studies support Pajor et al. (1991) motivation or compensatory feeding hypothesis (de Passillé et al., 1989; Algers et al., 1990; Appleby et al., 1992; Fraser et al., 1994; Huting et al., 2017). However, other studies did not observe a relationship between creep feed consumption and birth weight (Bruininx et al., 2004; Collins et al., 2013) or teat order (Appleby et al., 1991; Delumeau and Meunier-Salaün, 1995). Bruininx et al. (2001) finds that lighter pigs (6.7 kg) had more daily visits to the feeder, but a lower feed intake per visit compared to heavier pigs (9.3 kg) PW. However, the study indicates that the lighter piglets ate more than middle-weight or heavy-weight pigs during the first 3 d PW. Which is in contrast to what Mahan et al. (1998) found. In general, the individual milk production of sows seems to be of greater importance for piglet gain than piglet birth weight or teat order, and therefore this is an unavoidable confounder when conducting research in creep feed. Hence, feed consumption or feed disappearance may be a better indicator of successful creep feeding.

### Diet Complexity

It was hypothesized that the complexity of the creep feed characterized by the composition of ingredients would be of minor importance in relation to piglet growth performance during lactation. As expected, we found no effects on feed disappearance and growth performance induced by diet complexity in the current study. The PCON and LCSP treatments did not result in different levels of feed disappearance, and the same was observed when the high and low complexity diets were provided in the form of large 12-mm pellets (HCLP and LCLP), even though aroma flavors such as vanilla and cheese had been added to the HCLP diet. The addition of aroma and/or flavor to creep feed to stimulate creep feed intake has been investigated in some studies (Yan et al., 2011a; Adeleye et al., 2014; Middelkoop et al., 2018). However, it is difficult to compare flavor preferences between studies with different experimental setups; even though red fruit was the flavor most preferred by piglets in the study by Middelkoop et al. (2018), it was the least preferred in the study by Adeleye et al. (2014). Although not significant, piglets fed LCLP and BMLP had a numerically higher feed disappearance compared with piglets fed large pellets with a high complexity. This could possibly be due to the lower protein content in the diet, since the bakery meal diet contained only whey protein and bakery meal, although litters fed LCLP showed the numerically highest creep feed disappearance. The higher feed disappearance may be explained by the BMLP piglets’ change from BMLP to LCLP on day 15. There was no difference in feed disappearance between the high complexity diets when the feed was provided in the form of small pellets or crumble, although piglets preferred a low complexity diet with large pellets compared with small pellets. The results for the current study partly support that a low complexity diet fed as large pellets increases the feed disappearance, as observed by Collins et al. (2013). However, the impact of diet complexity on ADG remains variable across studies and might be difficult to compare due to the variation of ingredients in the diets.

The lack of a statistically significant effect of diet complexity on BW and BW gain in the present study is consistent with the findings of Isensee et al. (2020) and Okai et al. (1976), who also found no difference in ADG during the pre-weaning creep feeding period when piglets were fed either a high or low complexity diet. However, previous studies have shown varying results regarding the impact of diet complexity on piglet growth performance and feed intake. The discrepancies between studies may be attributed to the difference in the level of complexity, as well as the timing of feed distribution, as mentioned previously. Fraser et al. (1994) and Heo et al. (2018) fed piglets a diet with either high or low complexity and found that the high complexity diet significantly improved ADG by 31 g and 61 g in week 4, respectively. On the other hand, Sands et al. (2021) compared the performance of piglets fed either a low or high complexity diet and found that piglets fed the low complexity diet had a 36 g higher ADG compared with piglets fed the low complexity diet in week 4 of lactation. In line with this, Collins et al. (2013) also observed a higher feed disappearance (+ 125 g/period) from days 19 to 25 of lactation and a tendency towards increased feed disappearance (+ 150 g/period) from days 26 to 29 in piglets fed a low complexity diet.

Although the term “diet complexity” is frequently used, it is not a specific term, which makes it difficult to draw comparisons between different trials. Diet complexity often refers to the inclusion of several high-digestibility protein sources and often also processed grains (e.g., popped wheat), but it can also refer to the diversity of ingredients, the addition of specific ingredients or specific functional properties (Menegat et al., 2022) or a high proportion of milk products (Mahan and Lepine, 1991). In the present study, high complexity diets included a variety of processed carbohydrate sources (e.g., corn flakes, wheat flakes, popped wheat and dehulled barley), and at the same time the protein sources covered a broad range of feedstuffs (e.g., soy protein concentrate, potato protein concentrate, skimmed milk powder, cheese powder, egg powder, and fish meal) and to a lesser extent soybean meal (only in PCON). The low complexity diets had a simpler composition and did not contain any processed carbohydrates except for 5% dehulled oats. Furthermore, the low complexity diets contained dehulled soybean meal (7%) to replace the more refined protein sources, although they still contained soy protein concentrate (5.73%), egg powder (2%), and fish meal (1%). It can be argued that our low complexity diets are medium complexity diets, but the omission of milk products (except for lactose) is still a major difference between the high and low complexity diets. Because the present study was conducted in a commercial herd without external fundings, it was not possible to test extreme dietary compositions, and furthermore the herd produced piglets for the UK market, which prohibits the use of blood meal and plasma in diets.

In recent studies, low complexity diets have contained as much as 34% soybean meal (Okai et al., 1976), whereas high complexity diets in earlier studies have been dominated by, for example, 42.1% whey protein (Heo et al., 2018) or 13.0% fish meal (Okai et al., 1976), or by providing diets based on 30.0% whey and animal protein sources such as meat meal and blood meal (Collins et al., 2013). This implies that there is no clear definition of low and high complexity. In the present study, several flavors and benzoic acid (0.50%), calcium formiate (0.70%), and prebiotics (Calsporin, Orffa, Breda, the Netherlands) as well as mycotoxin binders (Mycofix Plus, dsm-firmenich Animal Nutrition & Health, Kaiseraugst, Switzerland) were added to the high complexity diets. However, the low complexity diet did not contain any added flavors or prebiotics, although 1.0% mixed acid was included. Our high and low complexity diets differed in terms of both feedstuffs and feed additives, but this appeared to be of less importance than the pellet size.

### The Effect of Creep Feeding on Post-Weaning Performance

Piglets that did not receive creep feed during the lactation period had a numerically lower ADG (15% to 40%) from weaning to 15 days PW compared with piglets offered creep feed. Although previous studies have also reported no positive effect of creep feeding on PW performance (Isensee et al., 2020; Middelkoop et al., 2020; Christensen and Huber, 2021), in all of these studies, the piglets’ ADG was numerically improved when creep feed was offered during lactation. Other studies have reported that pre-weaning creep feeding increased the ADG PW (Fraser et al., 1994; Yan et al., 2011a; Adeleye et al., 2014; van den Brand et al., 2014; Craig et al., 2021). Creep feed offers an opportunity for piglets to become familiar with the ingestion of solid feed prior to weaning, and this might explain why they typically start eating more quickly PW. The lack of effect could be attributed to the low feed intake observed in the current study, which is most likely related to the early weaning of the piglets.

## CONCLUSION

Feed disappearance from day 8 of lactation until weaning was higher when 12-mm pellets were offered compared with 2-mm pellets or 2-mm crumble. The increased feed disappearance was supported by observations showing that more creep feed directed the eating behavior of piglets fed large versus small pellets. However, the increased feed disappearance in piglets fed larger pellets did not affect weaning weight or PW growth performance compared with piglets fed smaller pellets or piglets that did not receive creep feed. Diet complexity did not influence feed disappearance or growth performance, suggesting that the diet composition had less impact than the physical form of the pellets, at least when piglets were weaned at day 21.

## IMPLICATIONS

Although studies have shown varying results, further research should not only focus on productivity pre-weaning and PW but should also investigate health parameters, for example gut health and potential changes in microbiome caused by creep feeding. More studies should focus on the physical form of creep feed, since this appears to stimulate eating behavior and feed disappearance more than diet complexity. Additionally, studies involving piglets fed creep feed and weaned at four to five weeks of age may provide an insight into the long-term effects on creep feeding strategies. When piglets are weaned at three weeks of age, creep feeding primarily serves to stimulate eating behavior and help piglets adapt to solid feed. However, weaning at an older age may offer opportunities to influence weaning weight and, consequently, robustness at weaning.

## Confict of Interest Statement

Kristian Knage-Drangsfeldt is employed at Vestjyllands Andel but was not involved in the investigation. Thus, all authors declare that the trial was carried out in the absence of any conflicts of interest.
